# The role of echocardiography and ^99m^Tc-HDP scintigraphy in non-invasive diagnosis of cardiac amyloidosis: A case series and literature review

**DOI:** 10.1097/MD.0000000000017256

**Published:** 2019-09-20

**Authors:** Irina Iuliana Costache, Ana Maria Buburuz, Daniela Crisu, Ana Maria Statescu, Carmen Ungureanu, Viviana Aursulesei

**Affiliations:** a Department of Cardiology, “St. Spiridon” Emergency Clinical Hospital; b University of Medicine and Pharmacy “Grigore T. Popa”; c Department of Nuclear Medicine, “St. Spiridon” Emergency Clinical Hospital; d Department of Pathology, “St. Spiridon” Emergency Clinical Hospital, Iasi, Romania.

**Keywords:** 99mTc-HPD, bone scintigraphy, cardiac amyloidosis, echocardiography, non-invasive diagnosis

## Abstract

**Rationale:**

Cardiac amyloidosis, considered for the last years to be a rare disease, is one of the determinants of HFpEF. The non-specific clinical presentation and the difficulties related to endomyocardial biopsy have made cardiac amyloidosis an underdiagnosed clinical entity. Improvement of non-invasive diagnostic techniques and the development of new therapies increased clinical awareness for this form of restrictive cardiomyopathy. We here summarize echocardiography and ^99m^Tc-HDP scintigraphy findings in 6 cases of cardiac amyloidosis and review the literature data of this progressive and fatal cardiomyopathy.

**Patients concerns:**

The main clinical manifestations were fatigue, low exercise tolerance and edemas. The right heart failure symptoms usually dominated the clinical picture.

**Diagnoses:**

All cases were evaluated by echocardiography; 3 cases were further examined by bone scintigraphy and 4 cases a peripheral biopsy was performed. Electrocardiography showed low-voltage QRS complexes and “pseudo-infarct” pattern in the precordial leads, contrary to the echocardiographic aspect, which revealed thickening of ventricle walls. Biatrial dilation and diastolic disfunction were observed. Impaired systolic function was detected in advanced stages of the disease. ^99m^Tc-HDP scintigraphy revealed cardiac uptake of radiopharmaceutical and managed to confirm the diagnosis in 1 case of cardiac amyloidosis in which salivary gland biopsy was negative.

**Interventions:**

The treatment was based on managing fluid balance, with the mainstream therapy represented by diuretics. Neurohormonal agents, usually used in heart failure treatment were avoided, due to poor tolerance and worsening of disease course. The management of these 6 cases was challenging due to the refractory manifestation of congestive heart failure.

**Outcomes:**

During follow-up, 4 of the 6 patients from the current study died in the first year after the final diagnosis was established.

**Lessons:**

Nuclear imaging of cardiac amyloidosis has a revolutionary development nowadays. Bone scintigraphy presents promising results for identifying patients at early stages of disease and to differentiate between cardiac amyloidosis types. Further studies are necessary for the standardization of imaging protocol and development of non-invasive diagnostic tools, especially in assessing the response to treatment and disease progression, for which little is known.

## 1 Introduction

Amyloidosis is a systemic, progressive disease with multiple organ involvement caused by deposits of misfolded proteins which eventually form extracellular deposits. Cardiac amyloidosis determines an infiltrative-restrictive form of cardiomyopathy.^[1,2,3]^

There are many types of amyloidosis based on the affected protein, but more than 90% of the cardiac involvement is represented by light chain amyloidosis (AL) and transthyretin amyloidosis (ATTR).^[1]^ ATTR has 2 sub-types: an acquired “wild-type” form ATTR (ATTRwt) and a genetically transmitted form – mutant ATTR (ATTRm).^[1,4]^ The treatment and the prognosis of disease depend on the localization and the type of amyloid disease.

Diagnosis of cardiac amyloidosis is challenging due to unspecific clinical manifestations, such as low exercise tolerance and edemas.^[3,5,6]^ Other symptoms, such as fatigue or weight loss can be present in patients with CA and some of the clinical findings indicate a specific type of CA (e.g. polyneuropathy, carpal tunnel syndrome, spinal stenosis in >50% of ATTR; macroglossia, periorbital purpura, or nephrotic range proteinuria for AL).^[1,5]^

Endomyocardial biopsy (EMB) is the gold-standard, with an IIa indication in the American College of Cardiology guidelines for diagnosis of cardiac amyloidosis.^[7]^ The invasive technique is highly specific for cardiac involvement, but it is recommended to be used in trained centers due to low, but persistent risk of complications.^[7,8]^ Also, EMB brings insufficient data about amyloid extension, progression of disease, or differentiation between cardiac amyloidosis types.^[9]^ Additionally, invasive procedures are difficult to be performed in fragile and older adults.^[10]^ Peripheral biopsy (fat pat aspiration, gingival mucosa, rectal mucosa) has lower index of diagnosis in systemic amyloidosis, up to 80% of the cases of AL, and an even smaller rate for ATTR diagnosis.^[11]^

Cardiac scintigraphy using bone seeking radiotracers with planar and SPECT (single-photon emission computed tomography) imaging has proved a higher than 90% sensibility and specificity in early detection of CA and it shows capacity to differentiate between ATTR and AL types. Improvement and standardization of nuclear cardiac imaging techniques, together with the emergence of promising tools such as PET (positron emission tomography) imaging, show promising results in early and specific diagnosis of cardiac amyloidosis.^[12,13]^

Herein, we summarize the clinical, imagistic and pathological aspects of 6 cases diagnosed with cardiac amyloidosis and review the literature data in order to emphasize the insights of non-invasive diagnostic and treatment particularities.

## 2 Methods

### 2.1 Subjects

The study was carried out according to the principles outlined by the Declaration of Helsinki and has been approved by local ethical committee. We included 6 patients diagnosed with cardiac amyloidosis in our service between December 2013 and June 2018, that have signed informed consent for their clinical records to be used for research purposes at the time of admission.

### 2.2 Imaging techniques

All patients were clinical evaluated and had electrocardiography (ECG) and transthoracic echocardiography (TTE) examinations. TTE evaluation was performed by using a Vivid S70N (General Electric Healthcare) with standard 2D views, pulsed- and continuous-wave Doppler as well as tissue Doppler imaging. 3 of the 6 patients were examined by technetium-99m diphosphonate (^99m^Tc-HDP) scintigraphy, by using a Siemens E.cam Signature Series Dual Detector. All patients received 740 MBq of ^99m^Tc-HDP intravenously and were imaged 10 minutes after and 3 hours later radiotracer administration. Whole body images were acquired at a scan speed of 16 cm/minute using low energy high resolution collimators and were followed by planar images and SPECT images centered on the thorax.

### 2.3 Pathological examination

Biopsy of gingival mucosa was performed in 4 cases (subject 1–4) and revealed amyloid deposits for subject 2 and subject 3. The confirmation of amyloidosis was performed by nervous biopsy for subject 1. The fragment of biopsy was fixed in 10% neutral buffered formalin and embedded in paraffin. Four-micrometer-thick sections were obtained and stained by standard hematoxylin-eosin (HE). Amyloid fibrils were detected as amorphous eosinophilic deposits with HE. Congo red staining followed by polarized light microscopy revealed specific “apple-green” birefringence.

## 3 Results

### 3.1 Clinical presentation and outcomes

The demographic, clinical, and imagistic findings of the patients are shown in Table 1. In the study we included 3 males and 3 female patients with cardiac amyloidosis, ranging from 40 to 86 years old (mean 62.5 ± 16.14 years old). 1 patient (subject 5) was diagnosed with multiple myeloma and AL with lambda chains, and the other 5 patients had ATTR.

**Table 1:**
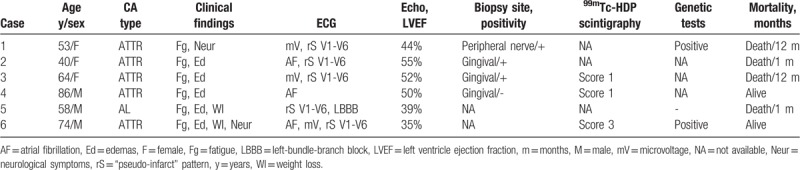
Demographic, clinical and imagistic characteristics of patients.

### 3.2 Electrocardiographic features

**Figure 1:**
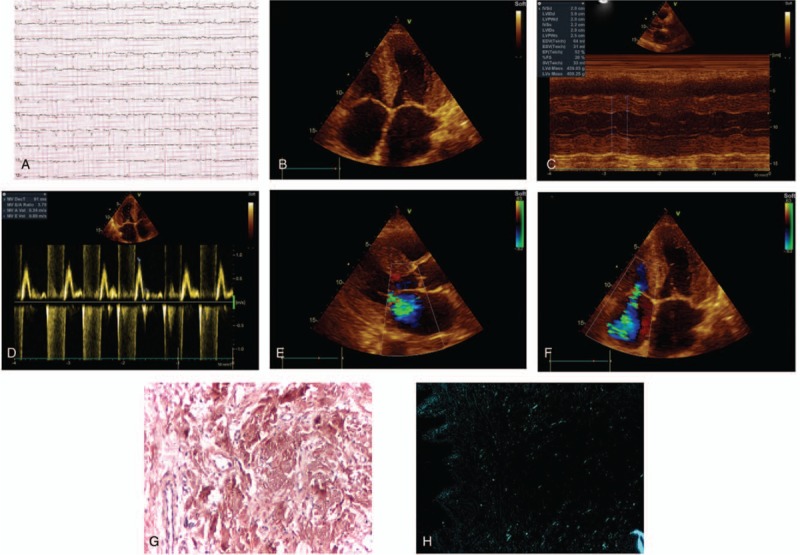
A 64-year-old female patient presented with dyspnea on small effort, fatigue, and edemas. Prior to present hospitalization she was diagnosed with heart failure and received diuretic treatment, but the symptoms persisted (subject 3).(A) ECG shows sinus rhythm, low-voltage QRS complexes in limb leads and slow progression of R wave in precordial leads (pseudo-infarct pattern). (B) 2D TTE apical 4-chamber view shows significative thickening of the ventricles, biatrial enlargement, thickening of atrioventricular valves, and interatrial septum. (C) 2D TTE M-mode shows preserved systolic function of left ventricle by Teicholtz method (LVEF 52%). (D) 2D TTE PW-doppler at mitral valve shows diastolic disfunction - E/A = 3.75 – restrictive filling of ventricles. E, 2D TTE parasternal long-axis view with color doppler – moderate mitral regurgitation. F, 2D TTE apical 4-chamber view with color doppler – severe tricuspid regurgitation. Salivary gland biopsy with Congo-red staining (10×) (G) shows the presence of amyloid deposits and under polarized-light microscopy (4×) (H) reveals apple-green birefringence of amyloid deposits.

ECG findings were analyzed and are recorded in Table 1. Slow progression of R wave in precordial leads, so called “pseudo-infarct” pattern and low-voltage QRS complex in the limb leads were observed (Fig. 1A). Presence of atrial fibrillation was diagnosed in 3 patients. Ventricle arrythmias, like premature ventricle beats or non-sustained ventricle tachycardia, were observed on Holter monitoring in 4 cases (subjects 3–6).

### 3.3 Echocardiographic features

We assessed echocardiographic changes highly suggestive for restrictive cardiomyopathy and cardiac amyloidosis, like impaired diastolic function with restrictive filling of the ventricles by pulsed-wave Doppler (PW-Doppler) and tissue-doppler imaging (TDI) (Fig. 1D), important symmetric thickening of left ventricle (≥15 mm) and biatrial enlargement (Fig. 1B and C). Evaluation of systolic function revealed that 3 cases associated mild-moderate systolic disfunction and the other 3 cases had preserved systolic function of the left ventricle (Fig. 1C). Thickening of the right ventricle (≥5 mm) was observed in 3 cases and small pericardial effusion was detected in 2 patients. Myocardial “granular sparkling” and thickening of the heart valves were observed in 4 cases (Fig. 1B), with secondary valve regurgitation (Fig. 1E and F), and interatrial septum was broader in subject 3 and subject 6.

### 3.4 Nuclear scintigraphy features

**Figure 2:**
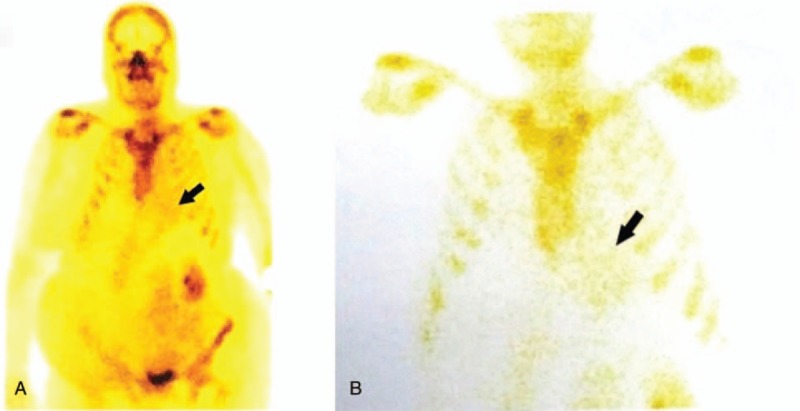
Bone scintigraphy was performed for subject 3 by intravenously administration of 740 MBq of ^99m^Tc-HDP. 3 hours later images were obtained. Mild (grade 1) cardiac uptake of radiopharmaceutical was seen in whole body image (A), planar static image of the thorax (B) (arrow).

^99m^Tc-HDP radiotracer cardiac uptake was graded using the semiquantitative visual score proposed by Perugini, based on comparative retention to the bone structure, where grade 0 = no cardiac uptake, grade 1 = mild cardiac uptake, lesser than bone, grade 2 = moderate cardiac uptake, similar to bone, grade 3 = high cardiac uptake, more than the bone.^[14,15]^ Subject 3 and subject 4 had grade 1 cardiac retention of ^99m^Tc-HDP (Fig. 2). Subject 6 had grade 3 cardiac uptake of ^99m^Tc-HDP.

### 3.5 Biopsy and pathology

Amyloid deposits from biopsy fragments of gingival mucosa were evaluated using standard HE and Congo red staining (Fig. 1G), also “apple-green” birefringence was observed by using polarized light microscopy (Fig. 1H). Subject 4 had negative gingival mucosa biopsy for amyloid. We did not perform endomyocardial biopsy due to the lack of equipment, low experience, and the associated risk of this invasive technique.

### 3.6 Genetic testing in ATTR

High index of suspicion, based on clinical and paraclinical results, for ATTR type lead to performing genetic tests for transthyretin gene mutations in 2 patients (subject 1 and subject 6), that were positive for transthyretin mutation. Even if the clinical suspicion of ATTR type was high and there were no proofs of AL, subject 2 and subject 4 did not benefit from genetic testing due to the lack of it at the moment of hospitalization and the early unfavorable outcome, with in-hospital death.

## 4 Discussion

Diagnosis of cardiac amyloidosis is frequently delayed by the belief to be a rare disease and the unspecific clinical presentation. In fact, increased clinical awareness and modern non-invasive diagnostic methods led to higher interest for identification of CA.^[1,9]^ Early diagnosis and specific treatment, based on amyloid type, induces better prognosis and decreases mortality.

The first key to diagnosis in cardiac amyloidosis is the mismatch between ECG aspect, with low QRS voltage or pseudo-infarct pattern in precordial leads, and transthoracic echocardiography with concentric thickened ventricle walls. Common echocardiographic findings are biatrial dilation and normal or reduced left ventricle dimensions.^[2,5,16]^ Diastolic disfunction appears from the early stages of the disease.^[8]^ Systolic function is preserved at the beginning, but in the late course of disease is decreased.^[3]^ “Sparkling” myocardium, given by amyloid deposits increased echogenicity, also can appear in the late stages of disease.^[3]^ Thickening of heart valves, of interatrial septum or of right ventricle walls are suggestive for CA, as a differentiation from other forms of restrictive cardiomyopathies.^[5]^ All the subjects included in our analysis had specific echocardiographic aspect of cardiac amyloidosis, consistent with the literature data. Pleural and small pericardial effusion can be manifestations of the disease.^[17,18]^ Also, development of strain imaging echocardiography, with specific pattern of “apical sparing”, is highly suggestive for the diagnosis of CA.^[1]^

Non-invasive methods, such as nuclear imaging techniques, have made a fundamentally change in cardiac amyloidosis evaluation. Over 30 years have passed since the incidental role of bisphosphonates in revealing cardiac amyloid burden on whole-body bone scans was demonstrated.^[9,12,19]^ Standardization of imaging protocol, taking into account radio-pharmaceutics for optimal image acquisition may improve the diagnosis, but even so, bone scintigraphy remains a valuable test for cardiac amyloidosis diagnosis.^[12]^

Nuclear imaging with bone tracers like ^99m^Tc-DPD (technetium-99m 3,3-diphosphono-1,2-propanodicarboxylic acid), ^99m^Tc-PYP (technetium-99m pyrophosphate) or ^99m^Tc-HDP can reach more than 90% specificity for CA, due to the high calcium levels in amyloid deposits, especially in ATTR.^[3,18,19,20]^ There are several debates over the use of one or another radiotracer, mainly related to the use of it in America or Europe, but also weight cost implications and availability.^[21–23]^

Improvement of nuclear techniques is considered to be revolutionary nowadays. Galat et al showed the role of early phase (soft tissue) image acquisition in predicting the late-phase (bone phase) findings by assessing heart to mediastinum cut-off ratio. This may be of use, especially in frail patients with renal disorder.^[24]^ Semi-quantitative evaluation by heart to whole body profile proved to be the most accurate ratio in determining cardiac amyloidosis by scintigraphy.^[25]^ Differentiating ATTR from other types of CA, or from normal population, based on myocardial uptake was emphasized by Ramsay et al. by quantifying the role of ^99m^Tc-HDP quantitative SPECT/CT in assessing a reference interval.^[26]^ Nevertheless, the role of bisphosphonates in evaluating disease progression or response to treatment was questioned. Casteno et al evaluated advanced ATTR patients and did not observe changes in myocardial uptake of ^99m^Tc-PYP, despite obvious disease progression.^[9]^ This is a small single center study on advanced ATTR cases and more research is necessary in the field of disease progression.^[27]^ Developing of modern techniques, such as PET (positron emission tomography) with agents like ^18^F-florbetaben shows promising results in evaluating amyloid burden, and distinguish between cardiac amyloidosis types.^[13,28,29]^

Bone scintigraphy can facilitate early diagnosis and make the difference between AL and ATTR types.^[10]^ Grade 0 and 1 in cardiac uptake of radiotracer are associated with AL and grade 2 and 3 cardiac uptake with ATTR.^[25]^ Gillmore et al demonstrated that the diagnosis of ATTR can be made by a Perugini visual score of 2 or 3 at scintigraphy in the absence of monoclonal protein in urine or plasma, without any histological proof.^[11]^ In our study, based on Gillmore et al demonstration, 1 patient (subject 6) was diagnosed with ATTR by bone scintigraphy visual score of 3, with following genetic positive test, avoiding invasive biopsy.

In our analysis, bone scintigraphy with ^99m^Tc-HDP managed to diagnose cardiac amyloidosis and was positive for subject 4, which had negative gingival biopsy for amyloid. This highlights the usefulness of nuclear imaging in early diagnosis of cardiac amyloidosis, with higher than 90% sensitivity and specificity for ATTR diagnosis, as it was demonstrated in the meta-analysis of Treglia et al.^[30]^

Treatment of cardiac amyloidosis involves heart failure management, with difficulties due to poor tolerance of neuro-hormonal agents, with the mainstream option represented by diuretic treatment.^[1,5,16]^ The management of cases included in the present study was also challenging due to the refractory manifestation of congestive heart failure. AL cardiac amyloidosis treatment is based on chemotherapy regimens aimed to eradicate proliferative plasma cell.^[9]^ Clinical trials emphasize the role of monoclonal antibodies in future treatment of AL. New opportunities emerged in ATTR treatment, such as transthyretin silencers (e.g. small interfering ARN - patisiran)^[28]^ and stabilizer of tetramer (e.g. diflunisal and tafamidis) that showed promising results. Other therapies are developing and have the potential of improved disease course.^[1,5,30]^ In advanced cases of cardiac amyloidosis, heart transplant remains the last option of treatment.^[1]^

During follow-up, 4 of the 6 patients of the current study died in the first year after the final diagnosis was established. This finding emphasis once more the poor prognosis of the disease. AL form has an even worse outcome, with median 6-months survival rate, compared to ATTR, for which is reported up to 24 to 66 months of survival, in the absence of specific treatment.^[1,5,31]^ Also, is important to highlight that ATTRm is autosomal dominant transmitted disease that requires genetic counseling.^[11,32,33]^

This study has some limitations. First, it is a retrospective analysis and not all the cases have complete medical investigations. Only 3 cases had ^99m^Tc-HDP scintigraphy and biopsy for amyloid deposits was performed from different sites in just 4 cases. Secondly, the limited number of patients and different form of cardiac amyloidosis included in the study need further investigation and additional series of cases to be evaluated, in order to determine the role of imaging techniques in diagnosis, prognosis assessment and future response to appropriate treatment.

## 5 Conclusions

^99m^Tc-HDP scintigraphy can represent a method of screening in early detection of cardiac amyloidosis and it is a useful tool in differentiating ATTR type from AL. Bone scintigraphy with myocardial uptake demonstrated a high accuracy for diagnosis, even if the results of peripheral biopsy are negative. Increased awareness of cardiac amyloidosis and the use of non-invasive imaging methods shorten the time to diagnosis and additionally improve outcome.

## Author contributions

**Conceptualization:** Irina Iuliana Costache, Ana Maria Buburuz.

**Data curation:** Ana Maria Buburuz, Daniela Crisu, Ana Maria Statescu, Carmen Ungureanu.

**Formal analysis:** Irina Iuliana Costache, Ana Maria Buburuz, Viviana Aursulesei.

**Project administration:** Irina Iuliana Costache, Ana Maria Buburuz, Daniela Crisu.

**Supervision:** Viviana Aursulesei.

**Writing – original draft:** Irina Iuliana Costache, Ana Maria Buburuz.

**Writing – review & editing:** Irina Iuliana Costache, Ana Maria Buburuz, Viviana Aursulesei.
